# Agroclimatology and Wheat Production: Coping with Climate Change

**DOI:** 10.3389/fpls.2018.00224

**Published:** 2018-02-20

**Authors:** Jerry L. Hatfield, Christian Dold

**Affiliations:** Supervisory Plant Physiologist and Research Associate, National Laboratory for Agriculture and the Environment, Agricultural Research Service, United States Department of Agriculture, Ames, IA, United States

**Keywords:** temperature, precipitation, yield gaps, agroclimatic indices, historical yields

## Abstract

Cereal production around the world is critical to the food supply for the human population. Crop productivity is primarily determined by a combination of temperature and precipitation because temperatures have to be in the range for plant growth and precipitation has to supply crop water requirements for a given environment. The question is often asked about the changes in productivity and what we can expect in the future and we evaluated the causes for variation in historical annual statewide wheat grain yields in Oklahoma, Kansas, and North Dakota across the Great Plains of United States. Wheat (*Triticum aestivum* L.) is adapted to this area and we focused on production in these states from 1950 to 2016. This analysis used a framework for annual yields using yield gaps between attainable and actual yields and found the primary cause of the variation among years were attributable to inadequate precipitation during the grain-filling period. In Oklahoma, wheat yields were reduced when April and May precipitation was limited (*r*^2^ = 0.70), while in Kansas, May precipitation was the dominant factor (*r*^2^ = 0.78), and in North Dakota June–July precipitation was the factor explaining yield variation (*r*^2^ = 0.65). Temperature varied among seasons and at the statewide level did not explain a significant portion of the yield variation. The pattern of increased variation in precipitation will cause further variation in wheat production across the Great Plains. Reducing yield variation among years will require adaptation practices that increase water availability to the crop coupled with the positive impact derived from other management practices, e.g., cultivars, fertilizer management, etc.

## Introduction

Agricultural ecosystems convert light, water, carbon dioxide, and nutrients into a variety of diverse plant products, e.g., carbohydrates, proteins, starch, etc. However, the changing climate, affects water availability, temperature, and atmospheric CO_2_ concentrations which in turn directly influences the plant growth processes and ultimately the ability of plants to efficiently produce the protein, starch, and other plant products that the human race requires as food. These effects are especially critical in cereal crops because of the importance in the human food supply. It is important to understand the role climate has on crop productivity and on individual plants and plant communities as part of agroecosystems.

Production variability in cereal crops in Queensland, Australia has been related to availability of precipitation and temperatures during the growing season (Yu et al., 2014). They found precipitation during the vegetative stage was the positive factor and most beneficial in determining grain yield, while exposure to high maximum temperatures depressed grain yields. Assessments of the future impacts of climate on agricultural productivity have been the subject of several recent summaries (Hatfield et al., 2011). These summaries have fostered extensive efforts to model the effects of future climate and have revealed that the continual increase in temperatures will depress wheat yields by 6% per °C increase (Asseng et al., 2015). Increasing carbon dioxide levels will increase growth; however, the positive effects are often offset by exposure to high temperatures and reduced precipitation (Hatfield et al., 2011). The Great Plains of the United States represent one of the most extensive areas of wheat (*Triticum aestivum* L.) production. Historical yields across the Great Plains provide an opportunity to evaluate the change in production relative to climate trends and to determine the effect of a changing climate on grain yields. One potential avenue to evaluate yield response is to examine the change in the yield gap, defined as the difference between the potential and actual yield. Licker et al. (2010) and van Brussel et al. (2015) have shown the value of yield gaps in being able to assess productivity in crops across the globe. Hatfield et al. (2017) utilized yield gap analysis across the Midwest for corn (*Zea mays* L.) and soybean [*Glycine max* (L.) Merr.] to determine the relationship between yield gaps and the meteorological conditions during the growing season. They found that July maximum, August minimum, and July–August precipitation totals were the dominant factors explaining yield gaps in these two crops across the Midwest. They utilized these relationships to estimate the potential impact of a changing climate across the Midwest and found with increasing temperatures and more variable summer precipitation there would be significant decreases in corn and soybean production.

**FIGURE 1:**
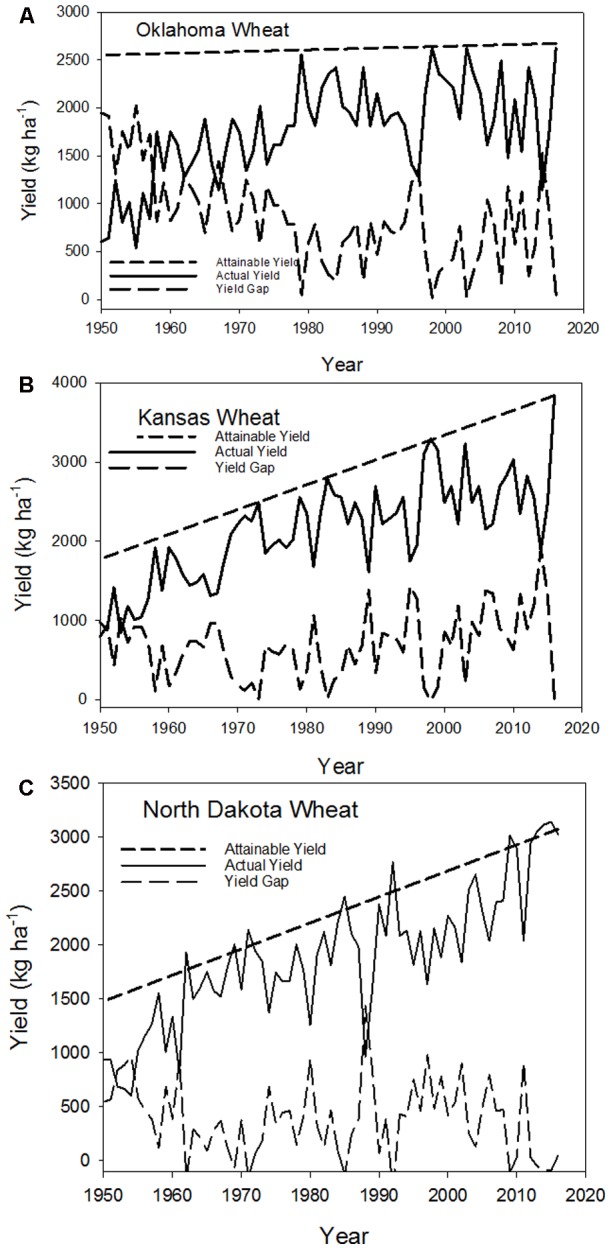
Attainable yield, actual yield, and yield gap for **(A)** Oklahoma, **(B)** Kansas, and **(C)** North Dakota statewide wheat yields since 1950 (data obtained from www.nass.usda.gov, accessed 2-December-2016).

Yield gap analysis was applied to the Great Plains of the United States using Kansas, Oklahoma, and North Dakota statewide yield data as examples of the changes in wheat productivity. These states were selected because Kansas and Oklahoma wheat yields at the state-wide production have shown a decline since 2000 with a recovery in yields in 2016 to near record levels (**Figure 1**). These trends are in contrast to wheat yields in in North Dakota that have continued to exhibit a yield increase with the typical annual variation due to variable weather during the growing season (**Figure 1**). Our goal was to evaluate the yield gaps in these three states and relate these yield gaps to the meteorological conditions during the growing season.

## Yield Gaps in Cereals

Throughout the history of agriculture, there has been the development of indices that describe how crops respond to the weather or how climate affects the distribution of crops around the world. Temperature and precipitation have been the two primary variables used in the development of these indices because of the availability of these data from public sources; however, the relationship of these indices to yield gaps has not been conducted. A recent study by Holzkämper et al. (2013) incorporated six factors into a crop suitability index that included average daily minimum temperatures below 0°C for frost impacts, daily mean temperature to determine plant growth, average daily maximum temperature above 35°C for heat stress, average daily soil water availability (precipitation–reference evapotranspiration), and length of the phenological period (days) to account for the effects of changing phenological development on biomass accumulation and crop yield. They were able to relate their index to maize yields for a number of locations around the world with a positive relationship between productivity and the suitability index. This approach is a refinement of the original approach by Neild and Richman (1981) to add more factors into their index to more closely match crop physiological responses.

Temperature impacts crop phenology and each species has a specific lower temperature value or base temperature, an optimum temperature value, and an upper temperature limit (Hatfield et al., 2011). Increases in temperature above the optimum have shown a negative impact on wheat yield with a projected 5.3% (Innes et al., 2015), and 6% (Asseng et al., 2015) yield reduction per 1°C rise. In wheat, exposure to frost or high temperatures during pollination has a significant effect on yield (Prasad and Djanaguiraman, 2014; Rezaei et al., 2015).

Adequate soil water supplies to the crop can offset the impacts of temperature extremes that are projected to increase during the growing season (Hansen et al., 2012; Collins et al., 2013; Walsh et al., 2014). These are difficult concepts to evaluate; however, understanding the linkage between historical yields and climate provides a foundation for future management scenarios.

To evaluate this framework, we computed the yield gaps for wheat production in Kansas, Oklahoma, and North Dakota following the approach of Egli and Hatfield (2014a) and Hatfield et al. (2017) using state level yield data since 1950. We selected 1950 as the beginning point in these analyses because this represents the agricultural era with modern technology. Yield gaps are computed as the difference between attainable yield, defined as the highest yields observed over the period of record, and the actual yield. Attainable yields are assumed to represent wheat yields under conditions that are non-limiting during the production year and a regression line is fit through these yields to obtain an attainable yield for each year. In this case study we used statewide yields rather than county yields to show the impact of climate variables at a large scale. It is evident for these three states that the attainable yield varies among states. For example, in Oklahoma, state level yields have shown only a modest increase since 1980 while Kansas and North Dakota have shown significant increases in grain production (**Figure 1**). Yield gaps for all three states showed variation from 1950 to 2016 and a statistical analysis of the yield gap with monthly maximum and minimum temperatures and precipitation observations was conducted. Regression analysis of monthly statewide average maximum and minimum temperatures and precipitation (data obtained from the Regional Climate Center) for the months of October, November, April, May, and June for Oklahoma and Kansas and April, May, June, and July for North Dakota against yield gaps for these three states revealed that precipitation was the only consistent and significant factor explaining yield gaps. For Oklahoma, the yield gap was explained by total April and May precipitation with a *r*^2^ = 0.7 and in Kansas the yield gap was due to May precipitation (*r*^2^ = 0.78). In North Dakota, with the later maturing crop, June and July precipitation was the dominant factor explaining 0.65 of the yield gap. Temperature for these three states showed no significant relationship to the variation in yield gap, even though there were years with temperatures that deviated from normal, these deviations were not sufficient to cause a change in statewide yields. Precipitation amounts below normal increased the yield gap and while low precipitation events are often associated with high temperatures, the phenology of the wheat crop with the grain-filling period earlier in the year reduces the potential for high temperature events. Although there were years in which the temperatures were above normal, these were not above the maximum temperature range for wheat for a significant period of time to become a significant factor reducing yield. Evaluating the effect of increasing temperatures has to account for the temperature increase relative to the temperature ranges of the crop. For example, Ahmed et al. (2017) showed an increase in wheat yields in the Pacific Northwest; however, these temperature increases are still within temperature ranges for the crop. We evaluated temperature effects using different temperature parameters for these data and found no consistent and significant relationships. This could be related to the fact that high temperature events or frost occur over short time periods, e.g., less than 5 days, and in more localized areas that are not detectable in monthly average data at the statewide scale, but can have significant impacts on local productivity (Prasad and Djanaguiraman, 2014; Rezaei et al., 2015). This does raise a caution about the scale being used in analysis of climate impacts on agriculture.

The primary inability to close the yield gap in the Great Plains was the lack of soil water to meet the water requirements of the wheat crop and insufficient precipitation amounts to recharge the soil profile during the grain-filling period. Egli and Hatfield (2014a,b) demonstrated that maize and soybean productivity were directly related to the ability of the soil to supply water during the grain-filling period. The dynamics of this response has been described by Hatfield (2012) to show the largest effect on maize yields in the central United States was the lack of sufficient water availability during the grain-filling period to meet the evaporative demand. The increase in precipitation variability with climate change will increase variation in crop yield (Hatfield et al., 2011). Soil water becomes the dominant factor affecting vegetative productivity in both cultivated and natural systems and the ability of the soil to infiltrate and store precipitation will become a critical factor to offset the impact of increasing variability in the changing precipitation regime. Increases in soil organic matter and the resultant impact on soil water holding capacity will increase the ability of a soil to store water and increase the infiltration rate. Both of these factors will increase the efficiency of a soil to offset variation in precipitation due to climate change.

**FIGURE 2:**
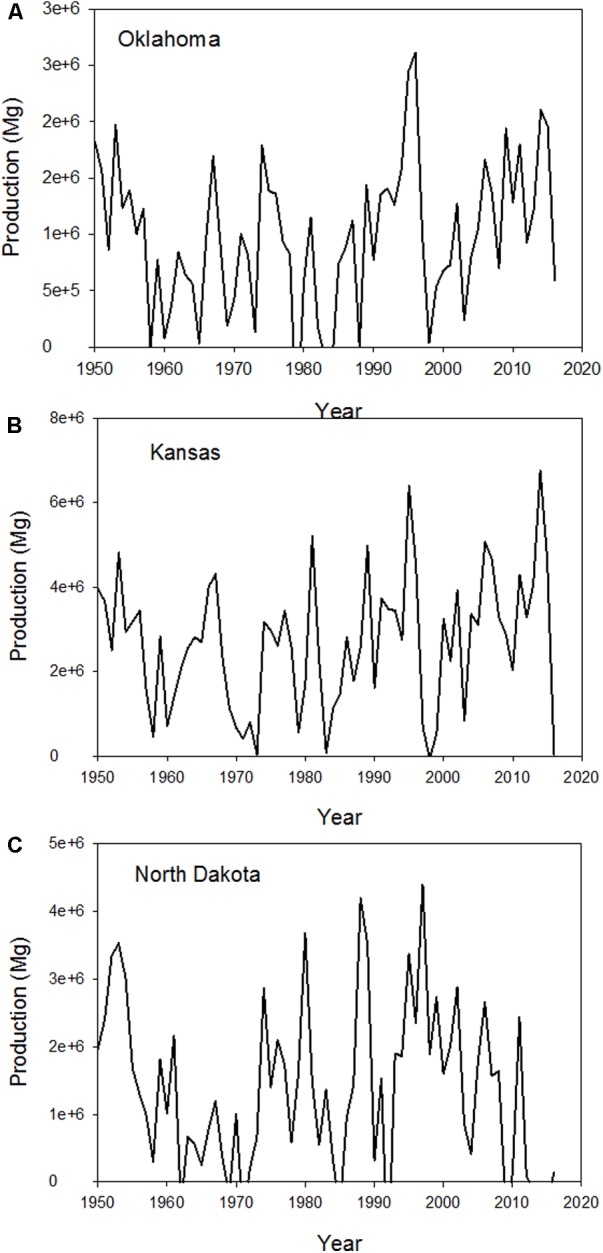
Production lost due to yield gaps occurring in **(A)** Oklahoma, **(B)** Kansas, and **(C)** North Dakota since 1950.

The magnitude of the yield gaps creates a large loss in wheat production across the Great Plains (**Figure 2**) and average about 20% of the attainable yield. The largest lost production in a given year was over 3 million Mg in Oklahoma, 6 million Mg in Kansas, and 4 million Mg in North Dakota during this period. This is a significant economic factor in each of these state economies. Since 1950, the production lost in these three states exceeds 65 million Mg in Oklahoma, 180 million Mg in Kansas, and 91 million Mg in North Dakota. These represent extremely large losses across the Great Plains and can be partially offset by management practices to increase climate resilience in our cropping systems. These management practices encompass how we manage the soil for water and nutrients, along with cultivar selection, and agronomic practices related to crop management for weeds, pests, and diseases.

## Coping With Climate Change

Variation in cereal production is directly linked with variations in precipitation and temperature and evident in the historical yield records. Projections of future changes in climate with warming temperatures and more variable precipitation will have impacts on crop productivity (Tao et al., 2009; Hao et al., 2013) and the recent analysis by Hatfield et al. (2017) for maize and soybean revealed that a combination of July maximum temperatures, August minimum temperatures, and July–August precipitation explained yield gaps across the Corn Belt. In this research study, we found for wheat in the Great Plains of the United States that precipitation was the dominant factor, with amounts during the grain-filling period the most critical in terms of affecting yield. Projections of precipitation for the critical months for wheat production in the Midwest obtained from https://climatetoolbox.org/tool/future-climate show that amounts will increase coupled with increased variation. We could expect yield variation among years to increase; however, the tendency to increase total amounts would suggest years with low yields may decrease leading to an overall increase in wheat productivity across the Great Plains until temperature increases become the dominant factor affecting grain yields. The projection that precipitation will become more variable during the spring months creates a situation in which management of soil water for the crop will be a necessary adaptation strategy to cope with climate change (Melillo et al., 2014). Protection of the soil resource to ensure available soil water will be critical to overcoming these impacts.

There is evidence in the literature to suggest that increasing temperatures will become more significant in affecting wheat productivity; however, some of this impact can be offset by ensuring these crops have an adequate soil water supply. Although, precipitation was a dominant factor in historical yields for these states, the recent results by Prasad and Djanaguiraman (2014), Tack et al. (2015), Karimi et al. (2017), and Kaur et al. (2017) suggest that we need to devote more attention to the effects of temperature on wheat productivity and suggest analyses and simulation models be utilized to evaluate the potential growing regions and productivity for wheat under future climate scenarios. To ensure continual advances in wheat productivity will require an integrated approach combining genetic improvement along with management practices and the approach we have outlined in the paper provides a framework for evaluating how we are progressing toward reducing the gap between genetic potential and actual yield.

## Author Contributions

This paper is a contribution from United States Department of Agriculture, Agricultural Research Service. JH wrote the paper and conducted the analysis. CD reviewed the materials and offered insights on the dynamics of environmental interactions.

## Conflict of Interest Statement

The authors declare that the research was conducted in the absence of any commercial or financial relationships that could be construed as a potential conflict of interest.

## References

[B1] Ahmed M. Stöckle C. O. Nelson R. Higgins S. (2017). Assessment of climate change and atmospheric CO_2_impact on winter wheat in the Pacific Northwest using a multimodel ensemble. *Front. Ecol. Evol.* 5:51 10.3389/fevo.2017.00051

[B2] Asseng S. Ewert F. Martre P. Rötter R. P. Lobell D. B. Cammarao D. (2015). Rising temperatures reduce global wheat production. *Nature Clim. Change* 5 143–147. 10.1038/nclimate2470

[B3] Collins M. Knutti R. Arblaster J. Dufresne J.-L. Fichefet T. Friedlingstein P. (eds) (2013). *Climate Change: The Physical Science Basis. Contribution of Working Group I to the Fifth Assessment Report of the Intergovernmental Panel on Climate Change*. Cambridge: Cambridge University Press, 108.

[B4] Egli D. B. Hatfield J. L. (2014a). Yield gaps and yield relationships in central U.S. soybean production systems. *Agron. J.* 106 560–566. 10.2134/agronj2013.0364

[B5] Egli D. B. Hatfield J. L. (2014b). Yield gaps and yield relationships in central U.S. maize production systems. *Agron. J.* 106 2248–2256. 10.2134/agronj14.0348

[B6] Hansen J. Sato M. Ruedy R. (2012). Perception of climate change. *Proc. Natl. Acad. Sci. U.S.A.* 109 E2415–E2423. 10.1073/pnas.1205276109 22869707 PMC3443154

[B7] Hao Z. AghaKouchak A. Phillips T. J. (2013). Changes in concurrent monthly precipitation and temperature extremes. *Environ. Res. Lett.* 8:034014 10.1088/1748-9326/8/3/034014

[B8] Hatfield J. L. (2012). “Spatial patterns of water and nitrogen response within corn production fields,” in *Agricultural Science*, ed. Aflakpui G. (Rijeka: Intech Publishers), 73–96.

[B9] Hatfield J. L. Boote K. J. Kimball B. A. Ziska L. H. Izaurralde R. C. Ort D. (2011). Climate impacts on agriculture: implications for crop production. *Agron. J.* 103 351–370. 10.2134/agronj2010.0303

[B10] Hatfield J. L. Wright-Morton L. Hall B. (2017). Vulnerability of grain crops and croplands in the Midwest to climatic variability and adaptation strategies. *Clim. Change* 146 263–275. 10.1007/s10584-017-1997-x

[B11] Holzkämper A. Calanca P. Fuhrer J. (2013). Identifying climatic limitations to grain maize yield potentials using a suitability evaluation approach. *Agric. For. Meteorol.* 168 149–159. 10.1016/j.agrformet.2012.09.004

[B12] Innes P. J. Tan D. K. Y. Van Ogtrop F. Amthor J. S. (2015). Effects of high-temperature episodes on wheat yields in New South Wales, Australia. *Agric. For. Meteorol.* 208 95–107. 10.1016/j.agrformet.2015.03.018

[B13] Karimi T. Stöckle C. O. Higgins S. S. Nelson T. L. Huggins D. (2017). Projected dryland cropping system shifts in the Pacific Northwest in response to climate change. *Front. Ecol. Evol.* 5:20 10.3389/fevo.2017.00020

[B14] Kaur H. Huggins D. R. Rupp R. A. Abatzoglou J. T. Stöckle C. O. Reganold J. P. (2017). Agro-ecological class stability decreases in response to climate change projections for the Pacific Northwest, USA. *Front. Ecol. Evol.* 5:51 10.3389/fevo.2017.00051

[B15] Licker R. Johnston M. Foley J. A. Barford C. Kucharik C. J. Monfreda C. (2010). Mind the gap: how do climate and agricultural management explain the ‘yield gap’ of croplands around the world? *Glob. Ecol. Biogeogr.* 19 769–782. 10.1111/j.1466-8238.2010.00563.x

[B16] Melillo J. M. Richmond T. C. Yohe G. W. (eds) (2014). *Climate Change Impacts in the United States: The Third National Climate Assessment*. Washington, DC: US Global Change Research Program, 462–486.

[B17] Neild R. E. Richman N. H. (1981). Agroclimatic normals for maize. *Agric. Meteorol.* 24 83–95. 10.1016/0002-1571(81)90035-2

[B18] Prasad P. V. V. Djanaguiraman M. (2014). Response of floret fertility and individual grain weight of wheat to high temperature stress: sensitive stages and thresholds for temperature and duration. *Funct. Plant Biol.* 41 1261–1269. 10.1071/FP14061 32481075

[B19] Rezaei E. E. Siebert S. Ewert F. (2015). Intensity of heat stress in winter wheat-phenology compensates for the adverse effect of global warming. *Environ. Res. Lett.* 10:024012 10.1088/1748-9326/10/2/024012

[B20] Tack J. Barkley A. Nalley N. N. (2015). Effect of warming temperatures on US wheat yields. *Proc. Natl. Acad. Sci. U.S.A.* 112 6931–6936. 10.1073/pnas.1415181112 25964323 PMC4460489

[B21] Tao F. Yokozawa M. Liu J. Zhang Z. (2009). Climate change, land use change, and China’s food security in the twenty-first century: an integrated perspective. *Clim. Change* 93 433–445. 10.1007/s10584-008-9491-0

[B22] van Brussel L. G. J. Grassini P. Van Wart J. Wolf J. Claessens L. Yang H. (2015). From field to atlas: upscaling of location-specific yield gap estimates. *Field Crops Res.* 177 98–108. 10.1016/j.fcr.2015.03.005

[B23] Walsh J. Wuebbles D. Hayhoe K. Kossin J. Kunkel K. Stephens G. (2014). “Ch. 2: Our Changing Climate,” in *Climate Change Impacts in the United States: The Third National Climate Assessment*, eds Melillo J. M. Richmond T. C. Yohe G. W. (Washington, DC: U.S. Global Change Research Program), 19–67. 10.7930/J0KW5CXT

[B24] Yu Q. Li L. Luo Q. Eamus D. Xu S. Chen C. (2014). Year patterns of climate impact on wheat yields. *Int. J. Climatol.* 34 518–528. 10.1002/joc.3704

